# Risk-adjusted CUSUM control charts for shared frailty survival models with application to hip replacement outcomes: a study using the NJR dataset

**DOI:** 10.1186/s12874-019-0853-2

**Published:** 2019-11-27

**Authors:** Alexander Begun, Elena Kulinskaya, Alexander J MacGregor

**Affiliations:** 1 School of Computing Sciences, University of East Anglia, Norwich Research Park, Norwich, NR47TJ UK; 2 Norwich Medical School, University of East Anglia, Norwich Research Park, Norwich, NR47TJ UK

**Keywords:** CUSUM charts, Baseline hazard function, Risk adjustment, Competing risks, Shared frailty, Bootstrap

## Abstract

**Background:**

Continuous monitoring of surgical outcomes after joint replacement is needed to detect which brands’ components have a higher than expected failure rate and are therefore no longer recommended to be used in surgical practice. We developed a monitoring method based on cumulative sum (CUSUM) chart specifically for this application.

**Methods:**

Our method entails the use of the competing risks model with the Weibull and the Gompertz hazard functions adjusted for observed covariates to approximate the baseline time-to-revision and time-to-death distributions, respectively. The correlated shared frailty terms for competing risks, corresponding to the operating unit, are also included in the model. A bootstrap-based boundary adjustment is then required for risk-adjusted CUSUM charts to guarantee a given probability of the false alarm rates. We propose a method to evaluate the CUSUM scores and the adjusted boundary for a survival model with the shared frailty terms. We also introduce a unit performance quality score based on the posterior frailty distribution. This method is illustrated using the 2003-2012 hip replacement data from the UK National Joint Registry (NJR).

**Results:**

We found that the best model included the shared frailty for revision but not for death. This means that the competing risks of revision and death are independent in NJR data. Our method was superior to the standard NJR methodology. For one of the two monitored components, it produced alarms four years before the increased failure rate came to the attention of the UK regulatory authorities. The hazard ratios of revision across the units varied from 0.38 to 2.28.

**Conclusions:**

An earlier detection of failure signal by our method in comparison to the standard method used by the NJR may be explained by proper risk-adjustment and the ability to accommodate time-dependent hazards. The continuous monitoring of hip replacement outcomes should include risk adjustment at both the individual and unit level.

## Background

Continuous monitoring of healthcare, and increasingly, social care across various providers is an important task of the healthcare regulator, such as the Care Quality Commission (QCC) in the UK. Additionally, a number of professional bodies and registers take on the same function for their clinical discipline. For instance, in regards to joint replacement, surgeon and operating unit-level outcomes are compiled by National Joint Registry for England and Wales (NJR). Methods of continuous monitoring of production quality have been initially developed and employed in quality control in the industry [1]. One of the most popular methods is the cumulative sum (CUSUM) chart, a graphical method based on sequential monitoring of cumulative performance over time. This method is based on sequential procedures and allows timely identification of a deterioration in performance. A number of CUSUM-based quality control systems are being implemented in various clinical disciplines, with the earliest application being in cardiothoracic surgery [2]. Currently they are used in surveillance of the healthcare quality by QCC [3], and by Dr Foster unit at Imperial College [4]. In this paper we expand the CUSUM methodology and adapt it for monitoring the performance of hip prostheses using the NJR data.

A hip replacement is a surgical operation where the damaged hip joint is replaced by a prosthesis. This operation is recommended to reduce pain and improve mobility of a patient after other therapies have failed. There are currently hundreds of types and brands of prosthesis components for use in the hip replacement surgery, and new brands of implant continue to be introduced through technological innovations. An important aspect of an implant brand’s performance is its expected time-to-revision. The current exception is that all prostheses used as treatment for end stage arthritis should have a failure rate of less than 5% at 10 years. Because of a relatively long time-to-failure of the hip prosthesis, long-term premarketing clinical trials are unfeasible. Therefore, continuous monitoring methods are needed for early detection of poor performance and timely withdrawal of the inferior components from clinical practice.

The first CUSUM-based methods for healthcare were based on binomial or Poisson distributions, monitoring failure rates within a fixed time interval, e.g. 30-days mortality [5], or one year hip replacement failure rates [6]. CUSUM methods for survival data are a natural extension of the methods for binary data. Censoring, truncation, adjustment for observed covariates and unobserved factors (frailties) can be easily included in survival models. By monitoring the individual-specific outcomes, the CUSUM score can be evaluated sequentially, changing at each individual failure. However, this method seems not to be appropriate in the case of hip replacement, where the expected time-to-revision is longer than 10 years. Hardoon et al. [7] proposed to compare the number of revisions within a certain time interval to that expected given a target revision rate and the total number of hip years in the interval. That is, patients contribute to a CUSUM score until revision or censoring (death or end of follow-up). They analysed the data from Swedish Arthroplasty register using Weibull distribution to model time to revision of hip replacement.

However, time-to-revision of hip prostheses varies depending on the patient characteristics, and on the type of fixation used [8]. This necessitates the use of case mix adjusted monitoring methods. The first risk adjusted CUSUM methods for time-to-failure (survival) data were introduced by Biswas and Kalbfleisch [9]. This method was picked up by the Scottish Arthroplasty Project, where CUSUMs are used to monitor complication rates of joint replacements by surgeon and unit from 2010. This is achieved by likelihood-based scoring method with risk adjustment for age, sex, osteoarthritis (OA) and rheumatoid arthritis (RA) [10]. A Bayesian-based CUSUM method for Weibull survival time is described in Assareh et al. [11].

Although the event of interest in our study is a revision, *a priori* death should not be treated as noninformative censoring. We develop a general competing risk version of the survival model for NJR data, where death is a competing risk. To safeguard the properties of the CUSUM charts, the control limits for risk-adjusted CUSUMs need to be revised to accommodate the estimation error.

We propose and implement a parametric version of the approach by Gandy and Kvaløy [12], of using bootstrap to provide the control limits conditional on the estimated in-control distribution, resulting in less conservative, i.e. more powerful, procedures.

We are using the Weibull distribution for fitting the baseline revision-specific hazard function, because this distribution has a good fit to the empirical distribution of time-to-revision [7]. The Gompertz distribution is used for fitting the baseline mortality-specific hazard function. The observed covariates and the correlated frailty components at the unit level are included in the model, assuming that all patients from a unit share the same unobservable gamma distributed risks of prosthesis revision and of death after hip replacement surgery.

We develop a bootstrap-based boundary adjustment for the risk-adjusted CUSUM chart to guarantee a given conditional probability of the false alarm rates. We also propose a score characterizing the quality of the hip replacement surgery in a unit. This score is based on the estimate of the posterior conditional frailties for units given the observed data. Mathematical development of the CUSUM scores for a Weibull/Gompertz survival model with shared frailty is provided in the Appendix.

The developed methods are applied to the 2003-2012 hip replacement data from the NJR. We illustrate the use of risk-adjusted CUSUM methodology to monitor the performance of two specific hip prostheses brands: the DePuy ASR Resurfacing Cup and the Biomet M2A-38 cup, which were flagged as outliers by NJR [13].

## Methods

### Motivating example

An artificial hip includes three major components: a stem that is inserted into the femur, a head (a ball) attached to the top of the femur and a cup, also called the acetabular component, that is implanted into the pelvis. A hip resurfacing procedure is typically used in younger patients where it can delay the need for a total hip replacement, it replaces the socket with an artificial cup and resurfaces the head of the femur instead of removing it. In 2010, NJR recorded 123 brands of acetabular cups, 13 brands of resurfacing cups and 146 brands of femoral stems used in primary and revision procedures [14].

Given a vast variety of available types and brands of prosthesis components for use in the hip replacement surgery, monitoring implant quality is the main objective of the NJR implant scrutiny group that was established in 2009. According to the current NJR methodology [15], an implant is considered to be a Level 1 outlier when its Patient Time Incident Rate (PTIR) is twice the PTIR of the implant group, where the group rate is weighted by the relevant implant types. From 2009 to 2014, three hip stems, three hip acetabular components and 17 hip stem/cup combinations were reported as Level 1 outliers [13].

To test our analytical approach on real world data, our analysis will focus on two of these outlier compoents: (i) the DePuy ASR Resurfacing Cup (first identified as a part of an outlier head/cup combination in April 2010 and last implanted in July 2010) and (ii) the Biomet M2A-38 acetabular cup (first identified by the NJR as an outlier in 2014, and last implanted in June 2011).

A standard CUSUM chart usually has a learning period where the parameters of the relevant null distribution are estimated, and the deviation from the null of clinical concern is decided upon to calibrate the control limits. The chart is then run with these control limits. An example of this approach is by Hardoon et al. [7], 2007 who monitored a constant target revision rate in a time interval. However, the failure rates differ by implant types, the age of the patients, and other case mix characteristics. They also may vary by the site at which operations take place (the operating unit). Therefore we consider a risk-adjusted CUSUM where the target rates are estimated for the popular implants (top 80%), and experienced units (more than 1 surgery per week, on average), which requires an introduction of shared frailty terms, describing similarities within and heterogeneity between units, to our survival models, and an appropriate adjustment of the control limits.

### Description of the NJR data

The NJR data were made available after a formal request to the NJR Research Committee. The dataset is related to the data cut used in the 10th NJR Annual Report [16]. The data were anonymised in respect to patient, to surgeon and to operating unit identifying details. Approval was obtained from Computing Subcommittee of the University of East Anglia Ethics Committee, reference number CMP/1718/F/10A. The NJR dataset provides the following four groups of variables used in the time-to-failure analysis of the hip replacements to risk-adjust the CUSUM boundaries.

- Information on procedures, such as date of operation or revision, and side;
- Institution and staff involved, such as unit and consultant IDs (anonymised), and surgeon grade;
- Hip prosthesis characteristics, such as fixation type (cemented, uncemented, hybrid, resurfacing), its components (head, cup, stem, and liner brands), head size, bearing surfaces (metal, polyethylene, ceramic);
- Patient characteristics, such as age, sex, ASA physical status classification [17] at 5 levels from healthy (1) to near death (5), Body Mass Index (BMI), index of multiple deprivation (IMD)[18] (a higher IMD means higher proportion of people in the area classed as deprived), and death date.

Since about a half of records had missing BMI values, this factor was excluded from further consideration. ASA scores were grouped into two categories in further analysis: ASA 1-2 - normal healthy patients and patients with mild systemic disease, ASA 3-5 - patients with serious, non-incapacitating systemic disease, patients with life-threatening incapacitating systemic disease and patients that are near death.

Data selection in SQL (elimination of duplicates, second and subsequent revisions) resulted in 504,024 records with the fields listed above. By further cleaning the following records have been additionally excluded:

- Patients with bilateral operations;
- Records with missing or misreported side;
- Records with time to revision equal to 0;
- Records with date of operation after 31 December 2012;
- Patients younger than 50 years at operation day;
- Records with missing values of IMD.

This process resulted in 281,265 records. Finally, all records for the patients operated in units with less than 52 operations per year (i.e. less than once per week, on average), and all records with implanted cup/head brands in the bottom 20% in popularity that year, as well as cup/head brands “DePuy” and “Biomet” were excluded in the in-control dataset, resulting in 113,772 records in total. To test the efficiency of our CUSUM procedure, we have also selected two test datasets including only the records with cup brands “DePuy ASR Resurfacing Cup” (1734 records) and “Biomet M2A 38” (764 records), respectively. The cases for prostheses revised within three months of implantation were censored at the time of revision to exclude failures that might be directly attributive to surgical technique or postoperative complications. Description of the three datasets is given in Table 1. We provide analysis of these data performed in R [19] in the “Results” section.

**Table 1:** Description of the datasets

Variable	Statistics	Control			DePuy			Biomet		
		Males	Females	All	Males	Females	All	Males	Females	All
Sample size	Number	44,468	69,304	113,772	1093	641	1734	315	449	764
	% by sex	39.1	60.9		63.0	37.0		41.2	58.8	
Revisions	Number	596	740	1336	132	169	301	15	36	51
	% by sex	44.6	55.4		43.9	56.1		29.4	70.6	
Deaths	Number	4074	5512	9586	56	31	87	40	37	77
	% by sex	42.5	57.5		64.4	35.6		51.9	48.1	
Censored	Number	39,798	63,052	102,850	905	441	1346	260	376	636
	% by sex	38.7	61.3		67.2	32.8		40.9	59.1	
Age	Mean	69.4	71.5	70.7	59.9	61.5	60.5	67.8	67.8	67.8
	StDev	9.1	9.3	9.2	6.9	8.5	7.6	7.3	7.6	7.5
IMD	Mean	19	19	19	18.6	17.3	18.1	11.6	12.3	12
	StDev	9.2	9.2	9.2	10.8	10.4	10.7	5	5.2	5.2
HeadSize	Mean	32.6	30.2	31.2	49.4	45.1	47.8	38	38	38
	StDev	6.5	3.8	5.2	3	2.6	3.5	0	0	0
Fixation										
Cemented	Number	18,787	36,150	54,937	71	35	106	8	7	15
	%	42.2	52.2	48.3	6.5	5.5	6.1	2.5	1.6	2
Uncemented	Number	13,522	17,679	31,201	503	403	906	297	434	731
	%	30.4	25.5	27.4	46	62.9	52.2	94.3	96.7	95.7
Hybrid	Number	9029	14,260	23,289	49	25	74	10	8	18
	%	20.3	20.6	20.5	4.5	3.9	4.3	3.2	1.8	2.4
Resurfacing	Number	3130	1215	4345	470	178	648	0	0	0
	%	7	1.8	3.8	43	27.8	37.4	0	0	0
ASA 1-2	Number	36,598	57,355	93,953	1012	587	1599	306	438	744
	%	82.3	82.8	82.6	92.6	91.6	92.2	97.1	97.6	97.4
ASA 3-5	Number	7870	11,949	19,819	81	54	135	9	11	20
	%	17.7	17.2	17.4	7.4	8.4	7.8	2.9	2.4	2.6
Cup/Head bearing surfaces										
Ceramic/Ceramic	Number	6584	8161	14,745	0	0	0	0	0	0
	%	14.8	11.8	13	0	0	0	0	0	0
Metal/Metal	Number	165	129	294	0	0	0	315	449	764
	%	0.4	0.2	0.3	0	0	0	100	100	100
Polyethylene/Ceramic	Number	4863	7070	11,933	0	0	0	0	0	0
	%	10.9	10.2	10.5	0	0	0	0	0	0
Polyethylene/Metal	Number	29,088	52,436	81,524	0	0	0	0	0	0
	%	65.4	75.7	71.7	0	0	0	0	0	0
Resurfacing/Metal	Number	318	233	551	534	447	981	0	0	0
	%	0.7	0.3	0.5	48.9	69.7	56.6	0	0	0
Resurfacing/Resurfacing	Number	3450	1275	4725	559	194	753	0	0	0
	%	7.8	1.8	4.2	51.1	30.3	43.4	0	0	0

### Basics of CUSUM method for time-to-event data

The CUSUM method is a sequential analysis technique based on the calculation of the series *W*_*i*_, *i*=0,1,2,..., defined by a simple recurrent equation

$$\begin{array}{@{}rcl@{}} \begin{aligned} W_{0} & = 0, \\ W_{i+1} & = \max \{0,\; W_{i}+X_{i}\}, \end{aligned} \end{array} $$

where index *i* stands for a single observation or for a group of observations and *X*_*i*_ is the weight or score assigned to index *i*. The CUSUM alerts when *W*_*i*_ crosses a control limit, usually chosen to guarantee a long average run length (ARL) when the process is in control, or to provide a low false alarm probability [20]. In applications to survival data, and assuming independent competing risks of revision and death, the score *X*_*i*_ for an individual *i* with time-to-revision *t*_*i*_ and vector of covariates **u**_*i*_ can be defined as the logarithm of the revision-specific factor of the likelihood ratio

$$\begin{array}{@{}rcl@{}} \begin{aligned} X_{i} = \log \left (\frac {f^{1}_{i}(t_{i}|\mathbf{u}_{i})^{\delta_{i}}S^{1}_{i}(t_{i}|\mathbf{u}_{i})^{1-\delta_{i}}}{f^{0}_{i}(t_{i}|\mathbf{u}_{i})^{\delta_{i}}S^{0}_{i}(t_{i}|\mathbf{u}_{i})^{1-\delta_{i}}}\right), \end{aligned} \end{array} $$

where *δ*_*i*_ is a censoring indicator, $S^{j}_{i}(.)$ and $f^{j}_{i}(.)$ are survival and density functions, respectively, and index *j*=0,1, stands for null hypothesis *H*_0_ (process is under control) and alternative hypothesis *H*_1_ (failure rate is higher than expected by a certain margin). Under the assumption of independent competing risks, the revision-specific factor of the likelihood coincides with the likelihood function that would be obtained be treating failures from any other causes as censored observations.

For a set *I* of independent individuals, the score *X*_*I*_ can be calculated as a sum of individual scores *X*_*i*_, *i*∈*I*:

$$\begin{array}{@{}rcl@{}} \begin{aligned} X_{I} = \sum_{i\in I} X_{i}. \end{aligned} \end{array} $$

Assuming proportional hazards model with the Weibull baseline distribution under hypotheses *H*_*j*_, *j*=0, 1, the hazard functions *h*_*j*_(*t*|**u**)=*μ*_*j*_(*t*)*χ*(**u**) are proportional to the Weibull baseline hazards *μ*_*j*_(*t*) and a regressor function *χ*(**u**). The regressor function is usually specified as *χ*(**u**)= exp(*β*^∗^**u**) (the Cox’s regression term) for a transposed column vector of unknown parameters *β*. The baseline hazard function under *H*_0_ corresponds to the hazard function *μ*_0_(*t*)=(*k*/*λ*)(*t*/*λ*)^*k*−1^ for the Weibull distribution with the shape parameter *k* and the scale parameter *λ*, and the baseline hazard function *μ*_1_(*t*) under the alternative hypothesis *H*_1_ is proportional to *μ*_0_, *μ*_1_(*t*)=HR*μ*_0_(*t*). The hazard ratio *HR* represents the departure from the target survival that we want to detect.

For consecutive time intervals *T*, consider a subset *I*=*I*_*T*_ of *N*_*I*_ individuals observed (prostheses in use) over the time interval *T*. In this case, the scores *X*_*I*_ can be calculated as [7]

$$\begin{array}{@{}rcl@{}} \begin{aligned} X_{I}=O_{I}\log(\text{HR})-(\text{HR}-1)E_{I}, \end{aligned} \end{array} $$

where *O*_*I*_ is the observed number of failures (revisions) occurring during the interval *T* and *E*_*I*_ is the number of failures that would be expected in the same interval under hypothesis *H*_0_.

Denote by (*t*_1*i*_,*t*_2*i*_) an intersection of the interval *T* with the lifetime of the prosthesis *i* implanted at *t*_0*i*_. Then *t*_1*i*_ is the maximum of the lower bound of interval *T* and *t*_0*i*_, and *t*_2*i*_ is the minimum of the upper bound of interval *T*, the time of revision of prosthesis *i* and the time of censoring of the patient with prosthesis *i*. From this, the value of (*t*_2*i*_−*t*_1*i*_) is equal to the length of time when prosthesis *i* is in use in the time interval *T*. The values of *E*_*I*_ can be computed as

$$\begin{array}{@{}rcl@{}} \begin{aligned} E_{I}=\sum_{i=1}^{N_{I}} \lambda^{-k}\left ((t_{2i}-t_{0i})^{k}-(t_{1i}-t_{0i})^{k}\right). \end{aligned} \end{array} $$

### CUSUM scores for shared frailty competing risks model

Under the proportional hazards model with frailty, the hazard functions *h*(*t*|**u**,*Z*) for an observed vector of covariates **u** and unobserved non-negative random frailty component *Z*, is proportional to the baseline hazard *μ*(*t*), frailty term *Z*, and a regressor function *χ*(**u**)= exp(*β*^∗^**u**). The conditional survival function is given by

$$ {\begin{aligned} S(t|\mathbf u, Z)\,=\,\exp(-\int_{0}^{t}h (x|\mathbf{u},Z)dx)=\exp(-Z\chi (\mathbf{u})\int_{0}^{t} \mu(x)dx). \end{aligned}}  $$

The marginal survival function is defined by

$$\begin{array}{@{}rcl@{}} \begin{aligned} S(t|\mathbf u)=\mathbb {E}S(t|\mathbf u, Z). \end{aligned} \end{array} $$

We will use the index *f*, *f*=*r*,*d*, to denote the types of failure (revision of implant or death of a patient without implant failure, respectively), considered as competing risks. For mathematical convenience, it is frequently assumed that frailty *Z*_*f*_ is gamma-distributed with mean 1 and unknown variance $\sigma _{f}^{2}$. The assumption of gamma distributed frailty is not too restrictive, as a number of authors demonstrated that gamma-based shared frailty models are robust for a wide class of frailty distributions [21, 22]. The frailty variance $\sigma _{f}^{2}$ characterizes heterogeneity in the population.

We also assume that the baseline hazard functions are $\phantom {\dot {i}\!}\mu _{0,r}(t)=(k_{r}/\lambda _{r})(t/\lambda _{r})^{k_{r}-1}$ and $\mu _{0,d}(t)=\lambda _{d}\exp (k_{d}t)\phantom {\dot {i}\!}$ with the shape parameter *k*_*f*_ and the scale parameter *λ*_*f*_, *f*=*r*,*d*, for the Weibull and Gompertz distributions, respectively. In this case, the type-of-failure specific marginal survival function is given by

$$\begin{array}{@{}rcl@{}} \begin{aligned} S_{f}(t|\mathbf u_{f})=(1+\sigma_{f}^{2}e^{\beta^{*}\mathbf u_{f}}H_{f}(t))^{-1/\sigma_{f}^{2}} \end{aligned} \end{array} $$

with the type-of-failure specific baseline cumulative hazards $H_{r}(t)=(t/\lambda _{r})^{k_{r}}\phantom {\dot {i}\!}$ and $\phantom {\dot {i}\!}H_{d}(t)=(\lambda _{d}/k_{d})(\exp (k_{d}t)-1)$.

Correlated frailty terms for revision and death can be constructed as

1 $$\begin{array}{@{}rcl@{}} \begin{aligned} Z_{r}= &Y_{0}+Y_{r}, \\ Z_{d}= &\frac {m_{r}}{m_{d}}Y_{0}+Y_{d} \end{aligned} \end{array} $$

for independent gamma distributed random variables *Y*_0_∼*G*(*l*_0_,*m*_*r*_) and *Y*_*f*_∼*G*(*l*_*f*_,*m*_*f*_) with $l_{f}=1/\sigma _{f}^{2}-l_{0}$, $m_{f}=1/\sigma _{f}^{2}$, *f*=*r*,*d*; 0≤*ρ*≤ min(*σ*_*r*_/*σ*_*d*_,*σ*_*d*_/*σ*_*r*_). The result of this construction is that the frailties are gamma-distributed with $\mathbb {E}Z_{f}=1$, $\text {Var}Z_{f}=\sigma _{f}^{2}$, and Corr(*Z*_*r*_,*Z*_*d*_)=*ρ*. Given the frailties (*Z*_*r*_,*Z*_*d*_) and the covariates (**u**_*r*_, **u**_*d*_), type-of-failure specific instantaneous risks are assumed to be conditionally independent at any time *t*.

The bivariate marginal survival function for the type-of-failure specific latent time moments (*t*_*r*_, *t*_*d*_) is given by the formula

$$\begin{array}{@{}rcl@{}} {\begin{aligned} S(t_{r},t_{d}|\mathbf u_{r},\mathbf u_{d})= &\mathbb {E}S(t_{r},t_{d}|\mathbf u_{r},\mathbf u_{d},Z_{r},Z_{d})  \\ =&\mathbb {E}\exp (-Z_{r}\chi (\mathbf u_{r})H_{r}(t_{r})-Z_{d}\chi(\mathbf u_{d})H_{d} (t_{d})) \\ = & \frac {\left(1+\sigma_{r}^{2}\chi (\mathbf u_{r})H_{r}(t_{r})\right)^{-l_{r}}\left(1+\sigma_{d}^{2}\chi (\mathbf u_{d})H_{d} t_{d}\right)^{-l_{d}}}{\left (1+\sigma_{r}^{2}\chi (\mathbf u_{r})H_{r}(t_{r})+\sigma_{d}^{2}\chi (\mathbf u_{d})H_{d}(t_{d})\right)^{l_{0}}}& \end{aligned}} \end{array} $$

[23]. If left truncation is present at ages (*t*_0*r*_, *t*_0*d*_), we calculate the conditional survival function by dividing the bivariate survival function by *S*(*t*_0*r*_,*t*_0*d*_|**u**_*r*_,**u**_*d*_).

In the context of hip replacement, the shared frailty terms arise from the assumption that the *n*_*j*_ patients who have undergone surgery in the same unit *j*, *j*=1,⋯,*J*, have the same, possibly correlated, unobserved risks of revision and death. This means that the full likelihood function for our model has a form of ${\mathcal L}=\prod _{j=1}^{J}{\mathcal L}_{j}(\bar t_{jr},\bar t_{jd}|\bar {\mathbf {u}}_{jr},\bar {\mathbf {u}}_{jd})$ for

2 $$\begin{array}{@{}rcl@{}} {\begin{aligned} &{\mathcal L}_{j}(\bar t_{jr},\bar t_{jd}| \bar{\mathbf{u}}_{jr}, \bar{\mathbf{u}}_{jd})=\prod_{i=1}^{n_{j}}\left (-\frac{\partial }{\partial t_{jir}}\right)^{\delta_{jir}}\left (-\frac{\partial }{\partial t_{jid}}\right)^{\delta_{jid}}S_{j}(\bar{t}_{jr},\bar t_{jd}| \bar{\mathbf{u}}_{jr},\bar{\mathbf{u}}_{jd}), \end{aligned}} \end{array} $$

where *δ*_*f*_=0,1 is the censoring indicator with *δ*_*f*_=0 indicating right censoring, and $\bar t_{jf}$ and $ \bar {\mathbf {u}}_{jf}$ are the vectors of cause-specific latent times and of covariates for the patients from unit *j*, respectively, *f*=*r*, *d*, and

$$\begin{array}{@{}rcl@{}} {\begin{aligned} &S_{j}(\bar t_{jr},\bar t_{jd}| \bar{\mathbf{u}}_{jr}, \bar{\mathbf{u}}_{jd}) \\ &=\frac {\left (1+\sigma_{r}^{2}\sum_{i=1}^{n_{j}}\chi (\mathbf u_{jir})H_{r}(t_{jir})\right)^{-l_{r}}\left (1+\sigma_{d}^{2}\sum_{i=1}^{n_{j}}\chi (\mathbf u_{jid})H_{d}(t_{jid})\right)^{-l_{d}}}{\left (1+\sigma_{r}^{2}\sum_{i=1}^{n_{j}}\chi (\mathbf u_{jir})H_{r}(t_{jir})+\sigma_{d}^{2}\sum_{i=1}^{n_{j}}\chi (\mathbf u_{jid})H_{d}(t_{jid})\right)^{l_{0}}}, \end{aligned}} \end{array} $$

where a subscript *i*, *i*=1,...,*n*_*j*_, corresponds to a current patient *i* from unit *j*. This likelihood can be used for parameter estimation.

Proposed CUSUM scores for a competing risks model with shared frailty are based on the likelihood ratio ${\mathcal L}$. For a time interval *T*, let *I*_*j*_(*T*) be a set of individuals from unit *j* whose implants are in use during the period *T*, and $I=I(T)=\bigcup I_{j}(T)$. The scores *X*_*I*_(*T*) for the time interval *T* are defined as

3 $$\begin{array}{@{}rcl@{}} {\begin{aligned} X_{I}(T) = \sum_{j =1}^{J}\log \left (\frac {\mathbb {E}\prod_{i \in I_{j}(T)}{\mathcal L}^{1}(t_{jir},t_{jid}|\mathbf{u}_{jir},\mathbf{u}_{jid},Z_{jr},Z_{jd})}{\mathbb {E}\prod_{i \in I_{j}(T)}{\mathcal L}^{0}(t_{jir},t_{jid}|\mathbf{u}_{jir},\mathbf{u}_{jid},Z_{jr},Z_{jd})}\right), \end{aligned}} \end{array} $$

where *Z*_*jr*_, *Z*_*jd*_ are the shared frailty terms for unit *j*, the superscript *h*, *h*=0,1, stands for hypothesis, and

$$\begin{array}{@{}rcl@{}} \begin{aligned} &{\mathcal L}^{h}(t_{jir},t_{jid}|\mathbf{u}_{jir},\mathbf{u}_{jid},Z_{jr},Z_{jd}) \\ &=\left (-\frac{\partial }{\partial t_{jir}}\right)^{\delta_{jir}}\left (-\frac{\partial }{\partial t_{jid}}\right)^{\delta_{jid}}S^{h}(t_{jir},t_{jid}|\mathbf u_{jir},\mathbf u_{jid},Z_{jr},Z_{jd}). \end{aligned} \end{array} $$

In general case, expression for *X*_*I*_(*T*) does not have a simple closed form. In the special case of *ρ*=0, the competing risks of revision and death are independent, and the score *X*_*I*_(*T*) is the sum of the respective component scores for revision and death (see Appendix). If the interest lies in the risk of revision only, death can be treated as a non-informative censoring, and we concentrate on the CUSUM analysis of revision scores to the end of this Section.

For the baseline Weibull hazard function, under the proportionate alternatives *μ*_1_(*t*)=HR*μ*_0_(*t*), we can rewrite the revision component of the score (3) as

4 $$\begin{array}{@{}rcl@{}} {\begin{aligned} &X_{I}^{r}(T) = O_{I}\log (\text{HR})-\sum_{j =1}^{J}({\sigma_{r}^{-2}}+O_{j})\\ &\times\log \left(\frac {1+\sigma_{r}^{2}\text{HR}\sum_{i \in I_{j}(T)}e^{\beta^{*}\mathbf u_{i}}\lambda^{-k}((t_{2i}-t_{0i})^{k}-(t_{1i}-t_{0i})^{k})}{1+\sigma_{r}^{2}\sum_{i \in I_{j}(T)}e^{\beta^{*}\mathbf u_{i}}\lambda^{-k}((t_{2i}-t_{0i})^{k}-(t_{1i}-t_{0i})^{k})}\right), \end{aligned}} \end{array} $$

where *O*_*j*_ is a number of revisions in the unit *j* during period *T* so that $O_{I}=\sum _{j}O_{j}$ (see Additional file 1 for proof).

Often, the proportional hazards assumption is too strong; different groups of patients and prostheses do not necessarily have proportional hazard functions for the hip revision times and/or for death. We weaken this assumption by allowing different shape parameters *k*_*f*_(**u**) in the baseline Weibull and Gompertz hazard functions which depend on covariates through additional Cox-regression multipliers, $k_{f}({\mathbf u})=\exp (\beta ^{*}_{k} \mathbf u)k_{f}$. Then the CUSUM scores for revision are calculated as

5 $$\begin{array}{@{}rcl@{}}  {\begin{aligned} &X_{I}^{r}(T) = O_{I} \log(\text{HR})-\sum_{j=1}^{J}(\sigma_{r}^{-2}+O_{j}) \\ &\times\log \left (\frac {1\,+\,\sigma_{r}^{2}\text{HR}\sum_{i \in I_{j}(T)}e^{\beta^{*}\mathbf u_{ji}}\lambda^{\,-\,k_{r}({\mathbf u_{ji}})}((t_{j2i}\,-\,t_{j0i})^{k_{r}({\mathbf u_{ji}})}\,-\,(t_{j1i}\,-\,t_{j0i})^{k_{r}({\mathbf u_{ji}})})}{1\,+\,\sigma_{r}^{2}\sum_{i \in I_{j}(T)}e^{\beta^{*}\mathbf u_{ji}}\lambda^{\,-\,k_{r}({\mathbf u_{ji}})}((t_{j2i}\,-\,t_{j0i})^{k_{r}({\mathbf u_{ji}})}\,-\,(t_{j1i}-t_{j0i})^{k_{r}({\mathbf u_{ji}})})}\right). \\ \end{aligned}} \end{array} $$

### CUSUM chart control limits for the shared frailty model for revision

The unknown parameters of the time-to-revision model under the null hypothesis *H*_0_ are estimated from the in-control (learning) dataset. These are the Cox-regression parameters *β* and *β*_*k*_, parameters of the Weibull baseline distributions *k* and *λ*, and the variance of the frailty term *σ*^2^. The vector of unknown parameters *ξ*=(ln*k*, ln*λ*, ln*σ*^2^,*β*,*β*_*k*_) is estimated using the maximum likelihood method to obtain the estimates $\hat \xi $. The time-to-failure distribution with these estimated parameters is then used to compute the CUSUM scores for the two test datasets and to estimate the control limits for the CUSUM chart: See Additional file 1 for details of calculation of the CUSUM score. Let *P*=*P*(*ξ*) be the true distribution function for revision times, and *τ*=*τ*_*c*_(*P*;*ξ*) is the time at which the chart alerts when it exceeds a threshold *c*. The false alarm probability in *T* time units is $hit(P;\xi) = \mathbb P(\tau _{c}(P;\xi) \leq T)$ for some finite *T*>0. The threshold *c*_*hit*_(*P*;*ξ*)= inf{*c*>0:*hit*(*P*;*ξ*)≤*α*} for some 0<*α*<1 is needed to restrict the false alarm probability to *α*. However, only $\hat P$ and $\hat \xi =\xi (\hat P)$ are known.

A parametric version of the bootstrap algorithm proposed by Gandy and Kvaløy [12] is used to estimate the control limits to guarantee, that the false alarm rate of a CUSUM chart with the in-control distribution *P*, conditional on $\hat \xi $, is below nominal level *α* with high probability 1−*γ*.

Define the first time $\tau _{c}(P|\hat \xi)$ at which the CUSUM chart conditional on $\hat \xi $ exceeds the given value *c*. We are interested in the boundary $c_{hit}(P|\hat \xi)$ defined by equation $c_{hit}(P|\hat \xi)=\inf \{c>0:\; \mathbb P(\tau _{c} (P|\hat \xi)\leq T)\leq \alpha \}$ for some 0<*α*<1. Since *P* is unknown, $c_{hit}(P|\hat \xi)$ is unknown too and the estimate $c_{hit}(\hat P|\hat \xi)$ is usually used instead. However, such estimate does not guarantee the false alarm rate of the chart. Following [12], we estimate the 1−*γ* quantile for the threshold $c_{hit}(P|\hat \xi)$ for some 0<*γ*<1 using the following algorithm.

*Algorithm.*

Let *N* be the number of records (patients) in the control dataset, *N*_*Sim*_ be the number of simulations needed to estimate $c_{hit}(\hat P|\hat \xi)$, *N*_*Boot*_ be the number of bootstrap replicates, and *T*=[*T*_*min*_,*T*_*max*_] be the observation period.

1. Calculate the maximum likelihood estimate (MLE) $\hat \xi $ of the vector of unknown parameters *ξ* as well as the estimate $\widehat {\text {Cov}}$ of the covariance matrix *cov* (inverse Hessian) for $\hat \xi $ using the control dataset and the survival model with Weibull hazard described above;
2. Generate from the multivariate normal distribution with mean $\hat \xi $ and the covariance matrix $\widehat {\text {Cov}}$, a random vector *ξ*_*cur*_;
3. Keeping the covariates in all three test datasets fixed, generate for all patients new times-to-revision *t*_*rev*_ on the basis of the survival model with Weibull hazard described above and vector *ξ*_*cur*_. Update the censoring using the rule *δ*=1 if *t*_*rev*_<= min{*t*_*death*_,*T*_*max*_} and *δ*=0, otherwise. Replace *t*_*rev*_ for *δ*=0 by *t*_*rev*_= min{*t*_*death*_,*T*_*max*_}. Repeat *N*_*Sim*_ times and calculate for the test dataset *j*, *j*=1,2, the values of $c_{\text {hit}}^{j}(\hat P_{cur}|\hat \xi _{cur})$ and $c_{\text {hit}}^{j}(\hat P|\hat \xi _{cur})$;
4. To take into account multiple testing, we set $c_{\text {hit}}(\hat P_{cur}|\hat \xi _{cur})=\underset {j=1,2}\max \{c_{\text {hit}}^{j}(\hat P_{cur}|\hat \xi _{cur})\}$ and $c_{\text {hit}}(\hat P|\hat \xi _{cur})=\underset {j=1,2}\max \{c_{\text {hit}}^{j}(\hat P|\hat \xi _{cur})\}$. Calculate $p_{cur}=c_{\text {hit}}(\hat P_{cur}|\hat \xi _{cur})-c_{\text {hit}}(\hat P|\hat \xi _{cur})$;
5. Repeat steps 2-4 *N*_*Boot*_ times and calculate the 1−*γ* empirical quantile *p*_*γ*_ of *p*_*cur*_.

The estimate of the adjusted threshold is equal to $c_{hit}(\hat P|\hat \xi)-p_{\gamma }$. This threshold guarantees that in approximately 100(1−*γ*)*%* of the applications the probability of false alarm will not exceed the value of *α*.In the “Results” section, we use the values of *N*_*Sim*_=100, *N*_*Boot*_=100, *α*=0.1, and *γ*=0.1, *T*_*min*_=01.01.2005, and *T*_*max*_=31.12.2012 for the analysis of the NJR data.

### Estimating operating unit performance

Estimating performance across surgical units is also of potential importance in the quality control setting. The posterior frailty distribution obtained from the fitted shared frailty survival model described in the “Methods” section, can be used for this purpose. Given the prior gamma distribution with (shape, scale) parameters (*a*,*b*)=(*σ*^−2^,*σ*^2^), mean *ab*=1 and variance *ab*^2^, and the observed data *D*_*j*_, the posterior frailty distribution for unit *j*, is the gamma distribution with (shape, scale) parameters (*a*_*j*_,*b*_*j*_) equal to

$$\begin{array}{@{}rcl@{}} \begin{aligned} a_{j}&=a+O_{j}, \\ b_{j}&=\frac {b}{1+b\sum_{i \in I_{j}}H(t_{i},\mathbf{u}_{i})}, \end{aligned} \end{array} $$

where *O*_*j*_ is the number of observed revisions in unit *j*, *I*_*j*_ is set of all patients from unit j, and *H*(*t*_*i*_,**u**_*i*_) is the cumulative hazard for individual *i* from unit j with time to revision (or censoring) *t*_*i*_ and the vector of covariates **u**_*i*_ [24].

The effects of the units (shared frailties) are given by the conditional expectation $\mathbb {E}(Z_{j}|D_{j})=a_{j}b_{j}$, and parameters *a*_*j*_ and *b*_*j*_ can be estimated by substituting the MLE estimates $\hat \xi $ of the unknown parameters *ξ* [21]. Given the proportional hazards formulation, the shared frailty term can be interpreted as an excess hazard of a unit relative to the baseline hazard. Because of this interpretation, we refer to these estimated frailties as unit-level hazard ratios and denote them by HR_*j*_.

Additionally, we propose a new score characterizing the quality of the hip replacement surgery in a unit as

6 $$ Q_{j}= P\{Z_{j}|D_{j}\} <1,  $$

where *D*_*j*_ is the data from the control dataset relating to unit *j*. Large value of *Q* indicates a decreased hazard of revision in a unit, whereas small value of *Q* indicates poor performance of a unit. Since the values of *Q* and HR depend on the vector of unknown parameters *ξ* and only the MLE estimate $\hat \xi $ of this vector is available, we generate a set of *N*_*average*_ estimates $\hat \xi _{l}$ from $\mathrm {N}(\hat \xi,\widehat {cov})$ distribution, and take the average of the obtained estimates of $Q(\hat \xi _{l})$ and of $\text {HR}_{j}(\hat \xi _{l})$ over this set of parameters.

## Results

For the control dataset described in the “Methods” section, we estimated unknown parameters of the competing risks model with and without shared frailty terms maximizing the likelihood function (2). These include the parameters for the baseline hazard distributions and the coefficients of the Cox’s regressions for time-to-revision and time-to-death, allowing for the possible covariate-dependent shape parameters, as described at the end of the “Methods” section. Significant predictors had been chosen using the backward elimination in stepwise regression. The estimated coefficients and their confidence intervals for the models with and without frailty components are given in Table 2. The notation $\phantom {\dot {i}\!}^{\prime \prime }{k}_{f}^{\prime \prime }$ before the name of a variable means that its coefficient relates to the shape parameter *k*_*f*_. The baseline values for the categorical and binary regressors were: males for sex, cemented for fixation, ceramic/ceramic for cup/head bearing surfaces, and operation date before 01.01.2007.

**Table 2:** Description, parameter estimates and confidence intervals for the competing risks models with/without frailty

Variable	No frailty terms		Frailty for revision only		Independent frailty terms		Correlated frailty terms	
Sample size	113,772		113,772		113,772		113,772	
Number of revisions	1336		1336		1336		1336	
Number of deaths	9586		9586		9586		9586	
Number of censored	102,850		102,850		102,850		102,850	
Loglik	-114132.6		-114081.9		-114081.9		-114081.9	
AIC	228305.2		228205.9		228207.9		228209.9	
BIC	228498.0		228408.3		228419.9		228431.6	
Revisions	Estimate	CI	Estimate	CI	Estimate	CI	Estimate	CI
*λ*_*r*_ (year)	15.46	8.923 - 26.79	11.58	7.298 - 18.39	11.52	7.262 - 18.27	11.59	7.298 - 18.4
*k*_*r*_	1.66	1.171 - 2.355	1.63	1.149 - 2.312	1.642	1.158 - 2.329	1.63	1.149 - 2.311
*k*_*r*_ Females	1.096	1.018 - 1.179	1.137	1.049 - 1.233	1.134	1.046 - 1.23	1.137	1.049 - 1.233
*k*_*r*_ Age	0.9936	0.9884 - 0.9988	0.9936	0.9884 - 0.9987	0.9934	0.9883 - 0.9986	0.9936	0.9884 - 0.9987
*k*_*r*_ Cup/Head Resurf/Metal	1.515	1.126 - 2.039	1.569	1.167 - 2.11	1.576	1.172 - 2.119	1.57	1.167 - 2.11
Operation Date from 2007	1.26	1.112 - 1.427	1.288	1.132 - 1.464	1.286	1.131 - 1.463	1.288	1.132 - 1.464
Age	0.9692	0.9621 - 0.9765	0.9695	0.963 - 0.976	0.9694	0.9629 - 0.9758	0.9695	0.963 - 0.976
Uncemented	1.732	1.522 - 1.97	1.595	1.376 - 1.85	1.595	1.376 - 1.85	1.595	1.376 - 1.85
Head size	0.9677	0.9506 - 0.985	0.9563	0.94 - 0.9728	0.9563	0.9401 - 0.9728	0.9563	0.94 - 0.9728
Cup/Head Poly/Ceram	0.6017	0.4859 - 0.745	0.6592	0.5275 - 0.8238	0.6592	0.5274 - 0.8238	0.6593	0.5276 - 0.824
Cup/Head Resurf/Metal	9.37	4.556 - 19.27	10.88	5.796 - 20.43	10.8	5.75 - 20.28	10.89	5.797 - 20.45
Cup/Head Resurf/Resurf	4.558	3.113 - 6.673	5.595	3.804 - 8.227	5.593	3.803 - 8.224	5.594	3.804 - 8.226
$\sigma ^{2}_{r}$	-	-	0.1829	0.1205 - 0.2778	0.1829	0.1205 - 0.2777	0.1837	0.121 - 0.2787
Deaths	Estimate	CI	Estimate	CI	Estimate	CI	Estimate	CI
10^5^ *λ*_*d*_ (1/year)	1.286	0.9214 - 1.795	1.286	0.9214 - 1.795	1.288	0.9223 - 1.8	1.284	0.9204 - 1.792
10*k*_*d*_ (1/year)	0.9873	0.9404 - 1.037	0.9873	0.9404 - 1.037	0.987	0.9398 - 1.037	0.9875	0.9406 - 1.037
*k*_*d*_ Operation Date from 2007	0.9624	0.9561 - 0.9687	0.9624	0.9561 - 0.9687	0.9624	0.9562 - 0.9688	0.9624	0.9561 - 0.9687
*k*_*d*_ ASA P3-P5	0.6133	0.5639 - 0.6671	0.6133	0.5639 - 0.6671	0.6135	0.5641 - 0.6672	0.6132	0.5638 - 0.6669
*k*_*d*_ Cup/Head Poly/Metal	0.9458	0.9073 - 0.9859	0.9458	0.9073 - 0.9859	0.9459	0.9073 - 0.9861	0.9456	0.9072 - 0.9857
ASA P3-P5	36.53	23.83 - 56.01	36.54	23.83 - 56.01	36.46	23.79 - 55.86	36.58	23.86 - 56.08
Cup/Head Poly/Metal	1.563	1.193 - 2.047	1.563	1.193 - 2.047	1.562	1.192 - 2.045	1.564	1.194 - 2.049
4-5 quintiles of the IMD	1.084	1.04 - 1.13	1.084	1.04 - 1.13	1.084	1.04 - 1.13	1.084	1.04 - 1.13
$\sigma ^{2}_{d} $	-	-	-	-	2.031e-07	2e-07 - 2e-07	5e-07	5e-07 - 5e-07
*ρ*	-	-	-	-	-	-	0.00166	0.0013 - 0.0020

Comparing likelihood, AIC and BIC values in Table 2 we see that the correlation between cause-specific frailties *Z*_*r*_ and *Z*_*d*_ does not differ significantly from zero, and the best (in terms of AIC and BIC) model includes a frailty term only for revision. That is, the risks of revision and death can be modelled as independent, and formula (5) can be used to calculate CUSUM scores for revision.

Females had a decreased hazard of revision of hip prostheses compared to males on the time-to-revision interval [ 0,*λ*]. Hazard of revision decreased with age and head size. Uncemented hip prostheses had an increased hazard of revision compared to cemented or hybrid fixation. The cup/head combinations with resurfacing/metal and resurfacing/resurfacing bearing surfaces also had increased hazards compared to other types of bearings, whereas the polyethylene/ceramic bearing surfaces provided a decreased risk of revision compared to the ceramic/ceramic ones. These results agree with the findings by [8]. Those patients who underwent the surgery after 01.01.2007 had an increased hazard of revision. This may reflect the fact that early revisions were missed by the NJR due to poor data quality in the early years. We also have found a significant random effect of units, with the estimated frailty variance $\sigma ^{2}_{r}$ equal to 0.18 with confidence interval of (0.12−0.28). i.e. the hazard of revision differed by units.

Patients with serious disease (ASA P3-P5) and patients from areas with high deprivation (IMD 4-5) had increased hazards of death. The cup/head combination with polyethylene/metal bearing surfaces had a significantly increased hazard of death compared to ceramic/ceramic bearing. The shape parameters for baseline hazards of death also differed by these factors and by the date of surgery before/after 01.01.2007.

**Fig. 1:**
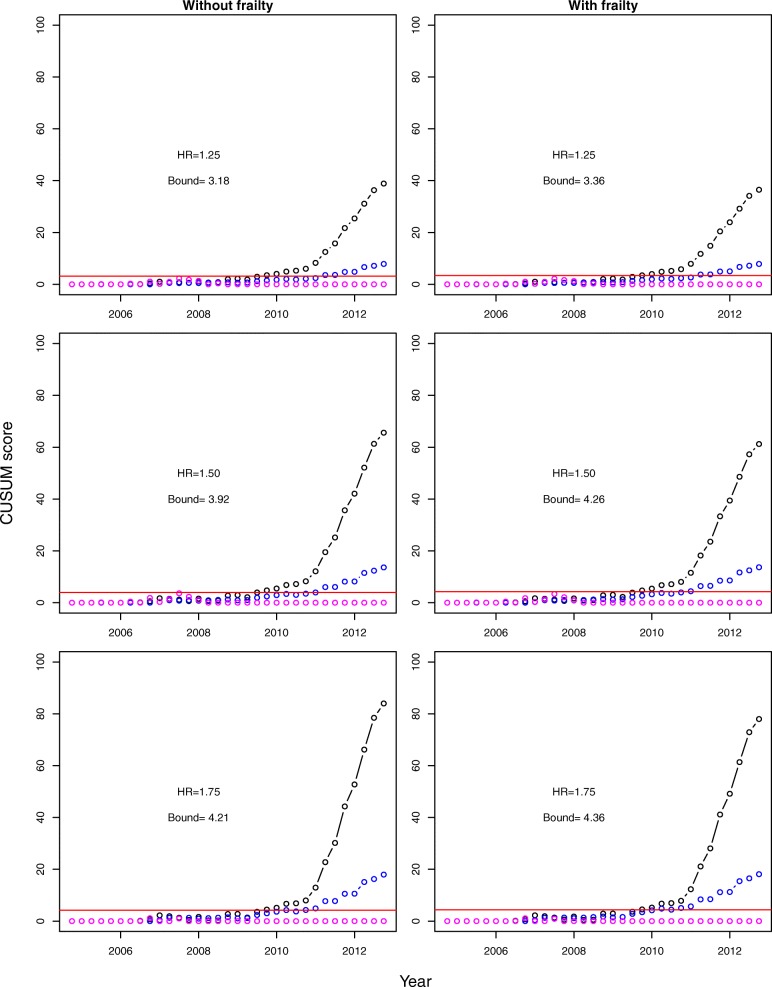
CUSUM charts calculated for quarterly revision rates in the three NJR datasets: DePuy ASR Resurfacing Cup (black), Biomet M2A 38 (blue) and in-control dataset (magenta), over the period 2005-12. The control bounds (solid red lines) are estimated by the parametric bootstrap

Based on the fitted revision submodel with frailty under independent competing risks, and targeting the hazard ratios of 1.25, 1.50 and 1.75 under alternative hypotheses, the CUSUM scores were calculated quarterly for the period 2005-12. The bootstrap-based boundaries were calculated at the false alarm rate *α*=0.1 and the tolerance level 1−*γ*=0.9 and adjusted for multiple comparisons for two tested hip implants. The CUSUM scores did not differ much between the models with and without frailty component. Figure 1 presents the CUSUM charts for the two test datasets as well as the in-control dataset for the models without/with frailty component at all three target hazard ratios. The CUSUM charts without frailty for DePuy ASR Resurfacing Cup produced alarm in the 4th quarter of 2009 for HRs of 1.25 and 1.75, and in the 3rd quarter of 2009 for HR of 1.50. The charts with frailty produced alarm somewhat later, in the 4th quarter of 2009 for all three values of the hazard ratio. This is comparable with the alarm based on PTIR by NJR in April 2010. For the Biomet M2A 38, the CUSUM charts without frailty hit the boundary in the second quarter of 2011 for HR=1.25, in the first quarter of 2011 for HR of 1.50, and in the second quarter of 2010 for HR= 1.75. The CUSUM charts with frailty alarm in the 2rd, the 1nd and the 2nd quarter of 2011, respectively. This is 3 to 4 years prior to the NJR alarm issued in 2014 [8].

The estimates of the quality scores *Q*_*j*_ and the hazard ratios HR_*j*_ have been calculated for 269 units included in the control dataset using *N*_*average*_=100. Our results demonstrate high heterogeneity in performance. 17 units out of the total of 269 had the quality scores greater than 0.9. HRs for these units were between 0.38 and 0.67. 15 units had the quality score values less than 0.1. Their HRs varied from 1.52 to 2.28.

**Fig. 2:**
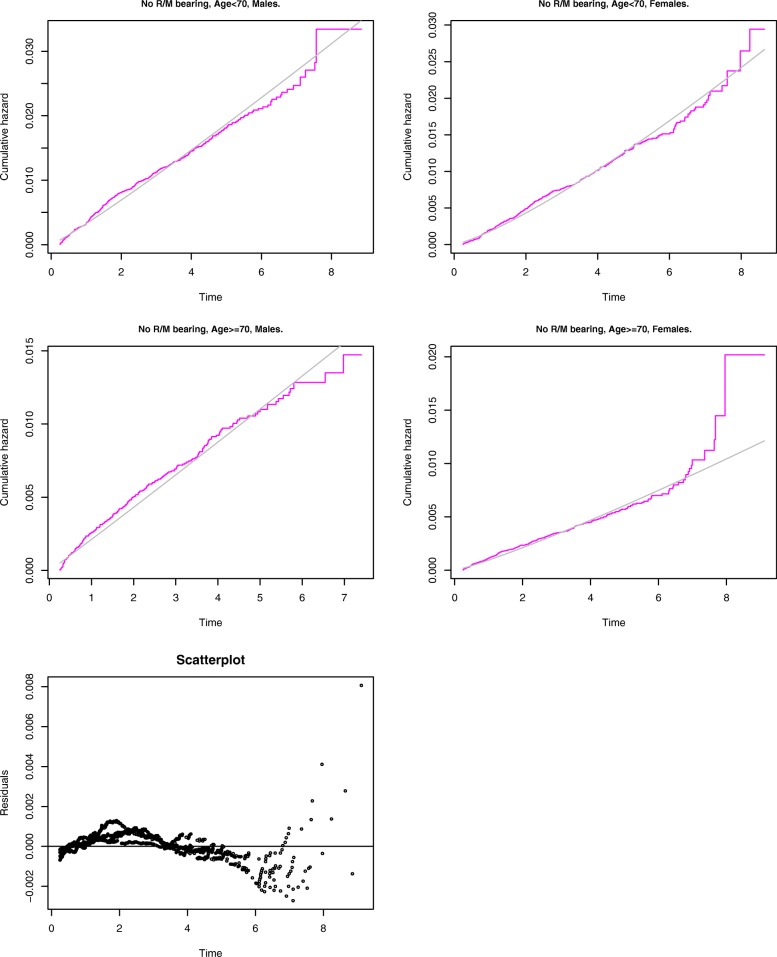
Comparison of the baseline cumulative hazard functions estimated using semiparametric (magenta) and parametric (Weibull model, grey) methods. Age&sex groups for revision data

**Fig. 3:**
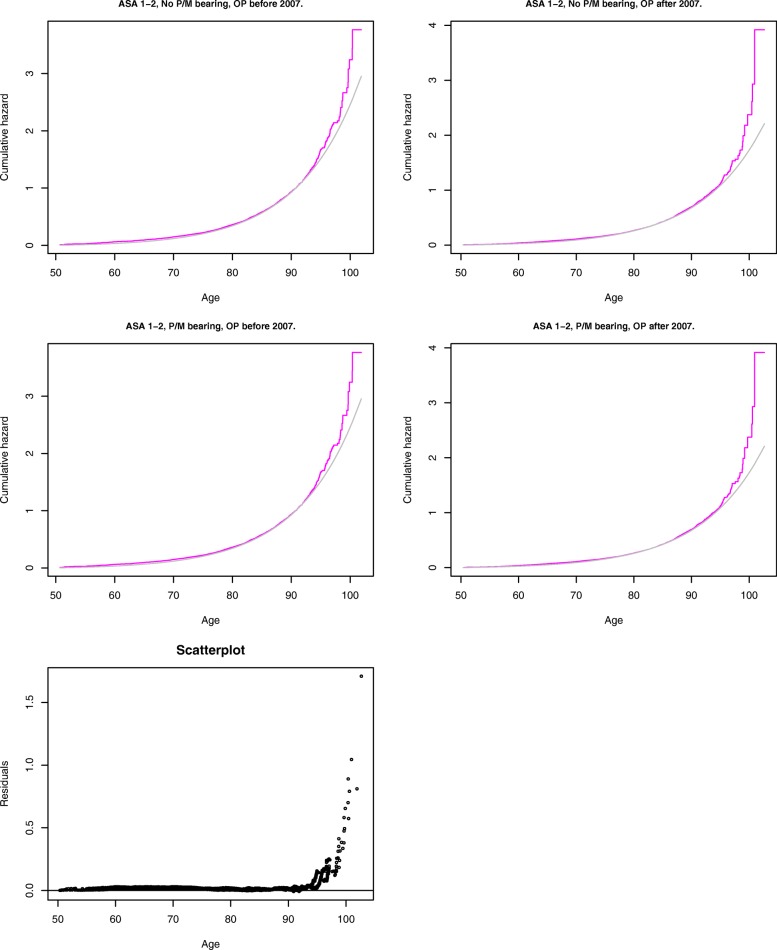
Comparison of the baseline cumulative hazard functions estimated using semiparametric (magenta) and parametric (Gompertz model, grey) methods. Date of operation & cup/head bearing groups for mortality data

To check the goodness-of-fit of chosen parametric distributions in our models for revision and mortality, we compared semiparametric estimates of baseline cumulative hazard functions to baseline cumulative hazards obtained from our parametric models, separately within each strata of a moderate to large size with a particular shape value. The results are shown in Fig. 2 for the Weibull baseline hazards in the revision model, and in Fig. 3 for the Gompertz baseline hazards in the mortality model. Additionally, these figures include plots of the residuals between the parametric and semiparametric estimates of the baseline hazards pooled across the strata. In Fig. 2, the larger deviations are still very small in absolute value, and mostly correspond to the small number of operations performed before 2007. Figure 3 is the confirmation of a well-known fact [25] that the Gompertz distribution describes human mortality well only up to 95 years, and the oldest patients in Fig. 3 are the outliers. Overall, the Weibull and the Gompertz models fit the revision and the mortality data, respectively, very well.

To assess the predictive value of our models, we also calculated the Harrell’s concordance index [26, 27] between the predicted and the observed survival. In the models without frailty, the estimates of the concordance were equal to 0.818 (*SE*=0.009) and 0.732 (*SE*=0.003) for revision and mortality data, respectively. For the models with frailty, the concordance values were equal to 0.819 (*SE*=0.009) and 0.732 (*SE*=0.003), respectively.

## Discussion

In hip replacement surgery, the continuous monitoring of the revision experience of hip prostheses is necessary due to delayed outcomes after the introduction of new brands into practice. CUSUM charts are a useful tool for early detection of changes in the revision rates after hip replacement. In the standard applications of the CUSUM-based monitoring, the learning data set required for the model identification is usually chosen from a preceding period. This assumes the stationarity of the process and leads to loss of information and the reduction of the period under study. Instead, we chose the in-control and the test data from the same period. This novel approach is especially beneficial for the future development of the adaptive version of the algorithm.

In the absence of the gold standard, the choices of the learning dataset and the model describing the data play an important role in the analysis using a self-starting CUSUM. After the routine cleaning of the original dataset, we excluded the records from units with less than 52 hip replacements per year to guarantee to some degree the sufficient experience of the implant within surgical teams. Similarly, only the top 80% of cup/head brands in each year were included to exclude rarely used brands, where the measure of failure rate was unlikely to be stable or robust.

Naive analysis treating competing risk events as noninformative censoring can lead to bias in estimates if competing risks are not independent. The competing risks model with dependent unobserved risk factors (frailties) is a convenient analytical tool for such data.

Two types of failure - revision and death without revision - are considered in this study. Other events during the follow-up period (e.g. loss to follow-up due to migration) are treated as noninformative censoring. In addition to observed factors, we included in the competing risks model correlating type-of-failure-specific random effects and all patients from a unit shared their values [28]. Sex, age, fixation, bearing surfaces, head size, and the date of operation were significantly associated with the life-time of the hip prosthesis. Bad health (ASA 3-5), high deprivation (IMD 4-5), polyethylene/metal bearing surfaces, and the date of operation were significantly associated with the higher hazards of death. These effects were robust against the frailty settings.

Identifiability of the competing risks model with random effects was studied in [29]. The main assumption for the identifiability of this model is the finite mean of the frailty. Identifiability of the bivariate survival models with time-dependent frailties given by the correlated Lévy-processes was studied in the recent publication [30]. Our methodology can be easily adapted to this scenario.

There is no consensus on whether the risks of revision and death are independent in hip replacement. Shwarzer et al. [31] showed these risks to be dependent in their data. However, a recent publication by Sayers et al. [32] argued for independence. Comparing the results from four competing risk models with and without shared frailty terms, we found that the best model included the shared frailty for revision but not for death. This means that the competing risks of revision and death are independent in the NJR data. The variance of the frailty term for revision differed significantly from zero, in other words, there were significant differences between units.

We used the classical AIC and BIC for the model selection. However, the conditional AIC (cAIC) [33–35] is more appropriate for use in frailty models, since the marginal AIC favors smaller models excluding random effects. We believe that the use of cAIC would not have changed our models because of the negligibly small values of the estimates for the variance of the frailty for mortality, the very small correlation between frailties, and the practically unchanged value of the log-likelihood compared to the model without a random effect for mortality. The cAIC methods are also very computationally intensive. However, our final model includes the random effect for revision. We intend to incorporate cAIC for model selection in our further work.

We proceeded with CUSUM monitoring of revision rates. The two cup brands, DePuy ASR Resurfacing Cup and Biomet M2A 38, were not included in the learning dataset and their performances were monitored using CUSUM charts. We calculated the adjusted boundaries for three target values of the hazard ratios to guarantee approximately 10% of false alarm rate with probability of 0.9 during the observation period 2005-12. The estimates of the boundary calculated using the models with the frailty component were higher, i.e. more conservative, than the one calculated using the model without the frailty component. This delayed two of the alarms, by three and by 12 months. The charts were comparatively robust to the changes in the target HR levels. The estimated CUSUM scores of the DePuy ASR Resurfacing Cup consistently increased from mid-2009. The increase of the CUSUM scores for the Biomet cup also started in 2009 and produced alarms in 2010-11, four years before the increased failure rate came to the attention of the UK regulatory authorities [15].

Estimating the posterior frailty distribution allows to compare the quality of the hip replacement surgery across units. From the 269 units included in the control dataset, 17 (6.3%) had a decreased hazard of revision with a quality score higher than 0.90 and 15 (5.6%) had an increased hazard of revision with a quality score less than 0.10. The associated hazard ratios of revision across the units varied from 0.38 to 2.28.

Due to low revision rates, the data set under study has about 90% censoring. The properties of the statistical methods in highly censored data sets are not well known. A further simulation study is required to assess the performance of our methods under varying amounts of censoring. Another limitation of this study is the choice of the gamma distribution for the correlated frailties. The advantage of the gamma frailty is a closed form expression for its Laplace transform. It allows for simple expressions for CUSUM scores. However, this choice results in necessarily positive correlations between revision and mortality frailties. Other forms of the frailty distributions (e.g. log-normal) to allow possible negative correlations will be pursued in our future work.

## Conclusions

This study developed and implemented, for the NJR data, continuous monitoring methods for surgical outcomes. We used the Weibull and the Gompertz hazard functions to describe the baseline hazards of revision and death, respectively. These functions appear to provide a good approximation to the respective type-of-failure life-time. However, adjustment for observed covariates is necessary to improve this approximation and to better understand the influence of the different factors on the life-times of the hip prosthesis and the patient.

Flexible parametrization taking into account possible influence of observed covariates on the shape and the slope parameters of the revision and mortality hazard functions as well as inclusion of the random effects (frailties) accommodate non-proportional hazards and improve the fit of our models to observed data.

Our results demonstrate that the competing risks of revision and death are independent in the NJR data. This finding will facilitate further development of continuous monitoring methods for these data.

We developed a novel method of CUSUM-based monitoring of revision rates. This method includes the choice of the in-control and the test data from the same period, and can be expanded for the subsequent development of an adaptive algorithm. Implementation of the special bootstrap algorithm to estimate the control limits in the CUSUM method guarantees with high probability that the false alarm rate is below a prespecified level. An earlier detection of failure signal by our method in comparison to the PTIR method may be explained by proper risk-adjustment and the ability to accommodate time-dependent hazards.

We found considerable variation in the hazard ratios of revision across the units. Therefore, the continuous monitoring of hip replacement outcomes should include risk adjustment at both the individual and unit level.

Our approach can be easily adapted to other practice areas requiring the continuous monitoring of the failure rates. Further development of the dynamic CUSUM-based methodology similar to that of [36] is needed to adapt our approach to real-time applications, where the new data are regularly updated. Additionally, more sophisticated methods are required to adjust for multiplicity if testing hundreds of various implant brands. We intend to address these further challenges elsewhere.

## Supplementary information

**Additional file 1** calculation of the CUSUM score

## Data Availability

The NJR data are available to interested researchers subject to approval of the data access request by the Healthcare Quality Improvement Partnership (HQIP) and governance controls. The R programs used to analyse the data are available from the authors on request.

## Abbreviations

ASA: American Society of Anesthesiologists
BMI: Body mass index
CUSUM: Cumulative sum
HR: Hazard Ratio
IMD: Index of Multiple Deprivation
NJR: National Joint Register
PTIR: Patient time incident rate
QCC: Care Quality Commission
