# Supplementary material for: Risk-adjusted CUSUM control charts for shared frailty survival models with application to hip replacement outcomes: a study using the NJR dataset
Source: BMC Med Res Methodol. 2019 Nov 27;19:217. doi: 10.1186/s12874-019-0853-2 (PMC6882343; doi:10.1186/s12874-019-0853-2)
Supplement: Supplementary file 1 — Additional file 1 calculation of the CUSUM score [file 12874_2019_853_MOESM1_ESM.pdf]

Additional file 1: Calculation of the CUSUM score

Preliminaries and notation

- Z_r and Z_d are gamma distributed frailties for revision and mortality in the competing risks model with proportional hazards;

- It is assumed that $\mathbb{E}Z_r = \mathbb{E}Z_d = 1$, $\text{Var}Z_r = \sigma_r^2$, $\text{Var}Z_d = \sigma_d^2$, and $\text{Corr}(Z_r, Z_d) = \rho$. The dependence between Z_r and Z_d is constructed through

$$Z_r = \frac{m_0}{m_r} Y_0 + Y_r,$$
$$Z_d = \frac{m_0}{m_d} Y_0 + Y_d,$$

  where Y_j are independent gamma distributed random variables, $Y_j \sim G(l_j, m_j)$, $j = 0, r, d$, with $l_0 = \rho/\sigma_r\sigma_d$, $l_j = 1/\sigma_j^{-2} - l_0$, $m_j = 1/\sigma_j^2$, $j = r, d$, $m_0 = m_r$, $0 \leqslant \rho \leqslant \min(\sigma_r/\sigma_d, \sigma_d/\sigma_r)$;

- Conditional bivariate survival function is given by

$$S(t_r, t_d|\mathbf{u}_r, \mathbf{u}_d, Z_r, Z_d) = \exp(-Z_r\chi(\mathbf{u}_r)H_r(t_r) - Z_d\chi(\mathbf{u}_d)H_d(t_d));$$

- Cause-specific cumulative function and the marginal survival function for latent time-to-failure t_f and the vector of covariates $\mathbf{u}_f$, $f = r, d$, are given by

$$H_f(t) = \int_0^t \mu_{0,f}(\tau)d\tau,$$
$$S_f(t|\mathbf{u}_f) = (1 + \sigma_f^2 e^{\beta^* \mathbf{u}_f} H_f(t))^{-1/\sigma_f^2}.$$

- Throughout, symbol "h" denotes a hypothesis, symbol "i" a patient, "j" a particular unit, and J is a number of units.

- Baseline hazard function for hypothesis H_h, $h = 0, 1$, is given by relation $\mu_{0,f}^h(t) = \text{HR}_f^h \mu_{0,f}(t)$, $f = r, d$.

- We define a cause-specific hazard ratio under hypothesis h, $H_f(h, t_f, \mathbf{u}_f) = HR_f \exp(\beta_f \mathbf{u}_f) H_f(t_f)$, $\tilde{H}_{jf,h} = 1 + \sigma_f^2 \sum_{i \in I_j(T)} H_f(h, t_{jif}, \mathbf{u}_{jif})$, $\tilde{H}_{j0,h} = \tilde{H}_{jr,h} + \tilde{H}_{jd,h} - 1$.

- For a time interval T, let $I_j(T)$ be a set of individuals from unit j whose implants are in use during the period T, and $I = I(T) = \bigcup I_j(T)$.
- $\binom{n}{k} = n!/(n-k)!k!$ is a binomial coefficient and $(a)_n = \Gamma(a+n)/\Gamma(a)$ is a Pohhammer's symbol.
- The numbers of revisions and deaths in a unit j during interval T are denoted by $O_{Ijr},\ O_{Ijd}$, respectively, and the totals are given by $O_{Ir} = \sum_j O_{Ijr}$ and $O_{Id} = \sum_j O_{Ijd}$.

Calculation of the score $X_I(T)$ in the general case of competing risks

The contribution $\mathcal{L}_j^h(T)$ of the period T to the likelihood is equal to

$$\mathcal{L}_j^h(T) = \mathbb{E} \prod_{i \in I_j(T)} \mathcal{L}^h(t_{jir}, t_{jid} | \mathbf{u}_{jir}, \mathbf{u}_{jid}, Z_{jr}, Z_{jd})$$

$$= \mathbb{E} \prod_{i \in I_j(T)} \left(-\frac{\partial}{\partial t_{jir}}\right)^{\delta_{jir}} \left(-\frac{\partial}{\partial t_{jid}}\right)^{\delta_{jid}} S^h(t_{jir}, t_{jid} | \mathbf{u}_{jir}, \mathbf{u}_{jid}, Z_{jr}, Z_{jd})$$

$$= (-1)^{O_{Ijr}}(-1)^{O_{Ijd}} \tilde{H}_{jr,h}^{-l_r} \tilde{H}_{jd,h}^{-l_d} \tilde{H}_{j0,h}^{-l_0} HR_r^{O_{Ijr}} HR_d^{O_{Ijd}}$$

$$\times (\sigma_r^2)^{O_{Ijr}} (\sigma_d^2)^{O_{Ijd}} \prod_{i \in I_j(T)} e^{\delta_{jir}\beta_{jir}\mathbf{u}_{jir}} e^{\delta_{jid}\beta_{jid}\mathbf{u}_{jid}} \mu_{0,r}(t_{jir})^{\delta_{jir}} \mu_{0,d}(t_{jid})^{\delta_{jid}}$$

$$\times \sum_{i_r=0}^{O_{Ijr}} \sum_{i_d=0}^{O_{Ijd}} \binom{O_{Ijr}}{i_r} \binom{O_{Ijd}}{i_d} (l_r)_{i_r} (l_d)_{i_d} (l_0)_{O_{Ijr}+O_{Ijd}-i_r-i_d} \tilde{H}_{jr,h}^{-i_r} \tilde{H}_{jd,h}^{-i_d} \tilde{H}_{j0,h}^{i_r+i_d-O_{Ijr}-O_{Ijd}}.$$

The score $X_I(T)$ for period T is given by

$$X_I(T) = \sum_{j=1}^{J} \log \left(\frac{\mathcal{L}_j^1(T)}{\mathcal{L}_j^0(T)} \right) \tag{1}$$

The case of independent competing risks

If revision and death are independent events ($\rho = 0$) then $l_0 = 0$, $(l_0)_k = 0$ for $k > 0$, and

$$\mathcal{L}_j^h(T) = \mathbb{E} \prod_{i \in I_j(T)} \left(-\frac{\partial}{\partial t_{jir}}\right)^{\delta_{jir}} S_r^h(t_{jir} | \mathbf{u}_{jir}, Z_{jr})$$

$$\times \mathbb{E} \prod_{i \in I_j(T)} \left(-\frac{\partial}{\partial t_{jid}}\right)^{\delta_{jid}} S_d^h(t_{jid} | \mathbf{u}_{jid}, Z_{jd}) = \mathcal{L}_{jr}^h(T) \mathcal{L}_{jd}^h(T),$$

where

$$\mathcal{L}_{jf}^{h}(T) = (-1)^{O_{Ijf}} \tilde{H}_{jf,h}^{-l_f - O_{Ijf}} HR_f^{O_{Ijf}} (\sigma_f^2)^{n_{jf}} (l_f)_{n_{jf}} \prod_{i \in I_j(T)} e^{\delta_{jif} \beta_{jif} \mathbf{u}_{jif}} \mu_{0,f}(t_{jif})^{\delta_{jif}}$$

for $f = r, d$. Formula (1) can be rewritten as

$$X_I(T) = \sum_{j=1}^{J} \log\left(\frac{\mathcal{L}_{j,r}^{1}(T)}{\mathcal{L}_{j,r}^{0}(T)}\right) + \sum_{j=1}^{J} \log\left(\frac{\mathcal{L}_{j,d}^{1}(T)}{\mathcal{L}_{j,d}^{0}(T)}\right) = X_{I,r}(T) + X_{I,d}(T),$$

where

$$X_{I,f} = \sum_{j=1}^{J} \log\left(\frac{\mathcal{L}_{j,f}^{1}(T)}{\mathcal{L}_{j,f}^{0}(T)}\right) = \sum_{j=1}^{J} \log\left(\frac{HR_f^{O_{Ijf}} \tilde{H}_{jf,1}^{-l_f - O_{Ijf}}}{\tilde{H}_{jf,0}^{-l_f - n_{jf}}}\right)$$

$$= \log(HR_f) \sum_{j=1}^{J} O_{Ijf} - \sum_{j=1}^{J} \log(\sigma_f^{-2} + O_{Ijf}) \left(\frac{\tilde{H}_{jf,1}}{\tilde{H}_{jf,0}}\right)$$

$$= O_{If} \log(HR) -$$

$$\sum_{j=1}^{J} (\sigma_f^{-2} + O_{Ijf}) \log\left(\frac{1 + \sigma_f^2 HR_f \sum_{i \in I_j(T)} e^{\beta_f^* \mathbf{u}_{jif}} (H_f(t_{j2if} - t_{j0if}) - H_f(t_{j1if} - t_{j0if})))}{1 + \sigma_f^2 \sum_{i \in I_j(T)} e^{\beta_f^* \mathbf{u}_{jif}} (H_f(t_{j2if} - t_{j0if}) - H_f(t_{j1if} - t_{j0if})))}\right)$$

$$= O_{If} \log(HR_f) -$$

$$\sum_{j=1}^{J} (\sigma_f^{-2} + O_{Ijf}) \log\left(\frac{1 + \sigma_f^2 HR_f \sum_{i \in I_j(T)} e^{\beta_f^* \mathbf{u}_{jif}} \lambda_f^{-k_f} ((t_{j2if} - t_{j0if})^{k_f} - (t_{j1if} - t_{j0if})^{k_f})}{1 + \sigma_f^2 \sum_{i \in I_j(T)} e^{\beta_f^* \mathbf{u}_{jif}} \lambda_f^{-k_f} ((t_{j2if} - t_{j0if})^{k_f} - (t_{j1if} - t_{j0if})^{k_f})}\right)$$

and (t_{j1if}, t_{j2if}) is an intersection of the interval T with the life time of the prosthesis i implanted at t_{j0f} (or with the life time of the respective patient if $f = d$).

Remark. If the shape parameter k_f depends on covariates in the form of $k_f(\mathbf{u}_f) = \exp(\beta_{kf}^* \mathbf{u}_f) k_f$, substitute $k_f(\mathbf{u}_f)$ for k_f in the above derivation to obtain

$$X_{I,f}(T) = O_{If} \log(HR_f) - \sum_{j=1}^{J} (\sigma_f^{-2} + O_{Ijf})$$

$$\times \log\left(\frac{1 + \sigma_f^2 HR_f \sum_{i \in I_j(T)} e^{\beta_f^* \mathbf{u}_{jif}} \lambda^{-k_f(\mathbf{u}_{jif})} ((t_{j2if} - t_{j0if})^{k_f(\mathbf{u}_{jif})} - (t_{j1if} - t_{j0if})^{k_f(\mathbf{u}_{jif})})}{1 + \sigma_f^2 \sum_{i \in I_j(T)} e^{\beta_f^* \mathbf{u}_{jif}} \lambda_f^{-k_f(\mathbf{u}_{jif})} ((t_{j2if} - t_{j0if})^{k_f(\mathbf{u}_{jif})} - (t_{j1if} - t_{j0if})^{k_f(\mathbf{u}_{jif})})}\right).$$

The full score $X_I(T)$ is an additive function of the partial scores and expression for $X_{I,f}(T)$ depends only on HR_f and does not depend on the hazard

ratio for the competing cause of failure. That is, if we are interested only in the changes of HR_r, we can carry out the CUSUM analysis based only on the values of $X_{I,r}(T)$.
